# Examining the Academic Trends in Neuropsychological Tests for Executive Functions Using Virtual Reality: Systematic Literature Review

**DOI:** 10.2196/30249

**Published:** 2021-11-24

**Authors:** Euisung Kim, Jieun Han, Hojin Choi, Yannick Prié, Toinon Vigier, Samuel Bulteau, Gyu Hyun Kwon

**Affiliations:** 1 Graduate School of Technology and Innovation Management Hanyang University Seoul Republic of Korea; 2 Department of Neurology College of Medicine Hanyang University Seoul Republic of Korea; 3 Laboratory of Digital Science of Nantes (LS2N) CNRS UMR6004 Nantes Université Nantes France; 4 CHU Nantes Psychiatry Department Nantes France; 5 INSERM U1246, SPHERE University of Nantes University of Tours Nantes France

**Keywords:** virtual reality, neuropsychological test, executive function, cognitive ability, brain disorder, immersive, digital health, cognition, academic trends, neurology

## Abstract

**Background:**

In neuropsychology, fully immersive virtual reality (VR) has been spotlighted as a promising tool. It is considered that VR not only overcomes the existing limitation of neuropsychological tests but is also appropriate for treating executive functions (EFs) within activities of daily living (ADL) due to its high ecological validity. While fully immersive VR offers new possibilities of neuropsychological tests, there are few studies that overview the intellectual landscape and academic trends in the research related to mainly targeted EFs with fully immersive VR.

**Objective:**

The objective of this study is to get an overview of the research trends that use VR in neuropsychological tests and to analyze the research trends using fully immersive VR neuropsychological tests with experimental articles.

**Methods:**

This review was carried out according to Preferred Reporting Items for Systematic Reviews and Meta-Analyses (PRISMA) guidelines. Articles were searched in three web databases using keywords related to VR, EFs, and cognitive abilities. The study was conducted in two steps, keyword analysis and in-depth systematic review. In the web database search from 2000 to 2019, 1167 articles were initially collected, of which 234 articles in the eligibility phase were used to conduct keyword analysis and a total of 47 articles were included for systematic review.

**Results:**

In keyword analysis, the number of articles focused on dementia including the keywords “MCI,” “SCD,” and “dementia” were highlighted over the period, rather than other symptoms. In addition, we identified that the use of behavioral and physiological data in virtual environments (VEs) has dramatically increased in recent studies. In the systematic review, we focused on the purpose of study, assessment, treatment, and validation of usability and structure. We found that treatment studies and uncategorized studies including presence and cybersickness issues have emerged in the recent period. In addition, the target symptoms and range of participants were diversified.

**Conclusions:**

There has been a continuously increasing interest in dealing with neuropsychology by using fully immersive VR. Target cognitive abilities have been diversified, as well as target symptoms. Moreover, the concept of embodied cognition was transplanted in this research area.

## Introduction

Virtual reality (VR) is a state-of-the-art technology at present. With this technology, we can create realistic worlds from the real world and generate artificial experiences in real time [1]. For the past decade, technological progress has enabled VR to become more popular through decreasing costs and increasing convenience.

With regard to defining mixed reality, Milgram and Kishino [2] suggested the concept of the reality-virtuality continuum. It is powerful to overview the dimension of reality. Considering the scope of our research, we partly adopted their perspective and focused on “immersion” to classify VR. Immersion in VR can be explained as the perception that is created by various stimuli that provide an absorbing environment so that users think they are physically in a virtual world [3]. The VR system can be divided into fully immersive and semi- or non-immersive, depending on the degree of immersion [4]. Fully immersive VR provides 3D displays (eg, head-mounted display [HMD]) that effectively make the user feel they are existing inside the virtual environment (VE) for the highest level of immersion. Non-immersive VR is based on a desktop with a flat-screen monitor with low interaction (eg, personal computer, tablet). With a large, curved monitor or projector, semi-immersive VR offers moderate immersion and interaction (eg, Kinect). Thomas et al [5], who studied the impact of immersion with a virtual avatar, said that the degree of immersion significantly influences the virtual body ownership and the feeling of presence. In addition, an enhanced effect of fully immersive VR on presence was reported by Waltemate et al [6]. In this study, we define fully immersive VR as a cover with up to a 360° screen-like Cave Automatic Virtual Environment (CAVE), dome-shaped screens, and HMDs [7].

Neuropsychological assessment is one way of examining the brain by its behavioral product [8]. Neuropsychological tests are typically used to assess/treat one’s cognitive abilities and further diagnose mental illness. By using neuropsychological tests, findable neuropsychological domains are extensive, for example, attention/concentration, language, visuospatial abilities, motor coordination, and executive functions (EFs) [9].

There are some popular traditional neuropsychological tests such as the Stroop test and the trail-making task [10,11]. These tests are widely accepted in professional society. Nevertheless, there are a few questionable limitations. One of the most critical limitations that is commonly pointed out is the lack of ecological validity. It is difficult to exactly measure the cognitive demands of real-world activities of daily living (ADL) with existing traditional neuropsychological tests [12,13].

As mentioned, the progress in VR technology gives rise to the possibility of applying existing neuropsychological tests in VR as an advanced model of the tests [9]. Pen-and-paper tests and computerized tests are widely used in neuropsychology, and they are composed of a set of predefined stimuli that exist in a controlled environment [14]. It is still necessary to increase ecological validity in measuring real-world performance or cognitive impairment [15-18]. However, VR environments can provide a realistic experience by multisensory stimulation and interactive factors as in daily life [19,20], with a strong sense of presence (“being there”) [21]; the means to test multitasking ability [22]; the influence of distractors that may not be used in a real-world laboratory [22]; and a good level of motivation of participants [23]. By using VR neuropsychological tests, it is possible to measure and evaluate cognitive abilities and EFs in a daily-living situation, even the instrumental activities of daily living (IADL) [24], and also, collecting behavioral data is enabled. IADL indicate activities using instruments that allow an individual to improve their quality of life, such as cooking, cleaning, and managing finance. There are many previous studies that have reported the specific connection between IADL and EFs [25-28]. These support that VR neuropsychological tests are significant, in that the IADL situation can be experimentally implemented through VR to measure or manage related cognitive functions. Therefore, VR can be considered an alternative or complementary innovative neuropsychological tool [29].

The term “executive function” was first defined by Muriel Deutsch Lezak in 1982 [30]. She said that “executive functions comprise those mental capacities necessary for formulating goals, planning how to achieve them, and carrying out the plans effectively” and proposed four classes as capacities of (1) formulating goals, (2) planning, (3) carrying out the plan, (4) and performing effectively [30]. However, the definition and functional categories of EFs are prescribed slightly differently by other researchers. Anderson [31] described EFs as “a collection of inter-related processes responsible for purposeful, goal-directed behavior” such as “anticipation, goal selection, planning, initiation of activity, self-regulation, mental flexibility, deployment of attention, and utilization of feedback.” Similarly, Hughes [32] described an EF as “a complex cognitive construct that encompasses the set of processes that underlie flexible goal-directed behavior (e.g., planning, inhibitory control, attentional flexibility and working memory).”

Therefore, there is a common agreement for categorizing three core EFs in general [33,34]: inhibition, working memory, and shifting (also called cognitive flexibility). Higher-order EFs, such as decision making (also called reasoning), problem solving, and planning, are usually established by extending from the core EFs [35,36]. Moreover, one of the core EFs, working memory, can be divided into more detail, such as general working memory, which holds information in the mind, and updating, which conducts translating instructions into action plans and involving new information into thinking or action plans [37].

In this paper, with advice from experts in neuropsychology based on the theoretical background, we categorized the EFs into seven sub-abilities:

- Inhibition: The ability to control impulsive and automatic responses and generate responses using attention and reasoning
- Working memory: The ability to temporarily store and handle information in order to do complex cognitive tasks
- Shifting: The ability to adapt your thoughts and behaviors to new, changing, and unexpected situations
- Decision making: The ability to efficiently and thoughtfully choose an option among different alternatives
- Problem solving: The ability to come to a logical conclusion when considering an unknown
- Planning: The ability to think about future events and mentally anticipate the correct way to carry out a task or reach a specific goal
- Updating: The ability to supervise behavior and ensure that you are properly carrying out the established plan of action

These days, fully immersive VR has been in the spotlight because it is considered an innovative tool to exceed the existing limitations of neuropsychological tests. Consequently, researchers who are studying in related areas have worked to improve the quality of VR and optimally apply it to neuropsychological tests. Many earlier studies have been conducted in the neuropsychological area with VR technology. However, there are only few studies that use fully immersive VR to mainly target EFs.

This systematic review article aims (1) to get an overview of the research trends using VR (including non-immersive) to conduct neuropsychological tests targeting EFs from 2000 to 2019 by keyword analysis and (2) to analyze the specific research trends using fully immersive VR with experimental articles.

## Methods

### Registration

This systematic review was conducted using the Preferred Reporting Items for Systematic Reviews and Meta-Analyses (PRISMA) guidelines [38]. In this study, we present a systematic review to summarize the past studies in the neuropsychological area with VR. Because there were few studies before 2000, the range of publishing years was limited from 2000 to 2019. The earlier studies were also significant and important. However, we assumed most of the concepts and topics in those studies could reflect on future studies, since there were a negligible number of studies that were related to VR and neuropsychological tests.

### Search Strategy

Initial articles were searched in three web databases: Scopus, Web of Science, and PubMed. These engines are adequate to cover the wide spectrum of topics in the target area. The articles were searched in March 2020, and the search query string was as follows: (“virtual reality” OR “VR” OR “virtual environment”) AND (“executive function(s)” OR “EF(s)” OR “cognitive function(s)” OR “cognition”). The publishing years were limited from 2000 to 2019.

### Inclusion Criteria

Journal articles and conference (proceeding) papers written in English were chosen, and other forms of publication, such as short reports, chapters of books, and dissertations, were excluded. To aim more deeply at EFs among the cognitive abilities, we included articles that were only related to “executive function” and “virtual reality” were used to see the effectiveness of neuropsychological tests in VR.

### Exclusion Criteria

After duplicates were removed, all studies were checked by using keywords and abstracts in the screening process. Review and protocol articles and chapters of books were all excluded. Moreover, articles not mainly focusing on EFs were excluded. At the end of the screening process, 234 studies were left.

Evaluation of full-text articles was conducted during the eligibility process. In this phase, we excluded theoretical articles, reporting articles, and non-experimental articles. The term “virtual reality” commonly corresponds not only to articles about immersive environments but also to articles about non-immersive environments, such as the use of a flat-screen monitor, so this study excluded them as not fully immersive VR articles. A few records where we could not find the full text were also excluded.

### Study Selection

**Figure 1:**
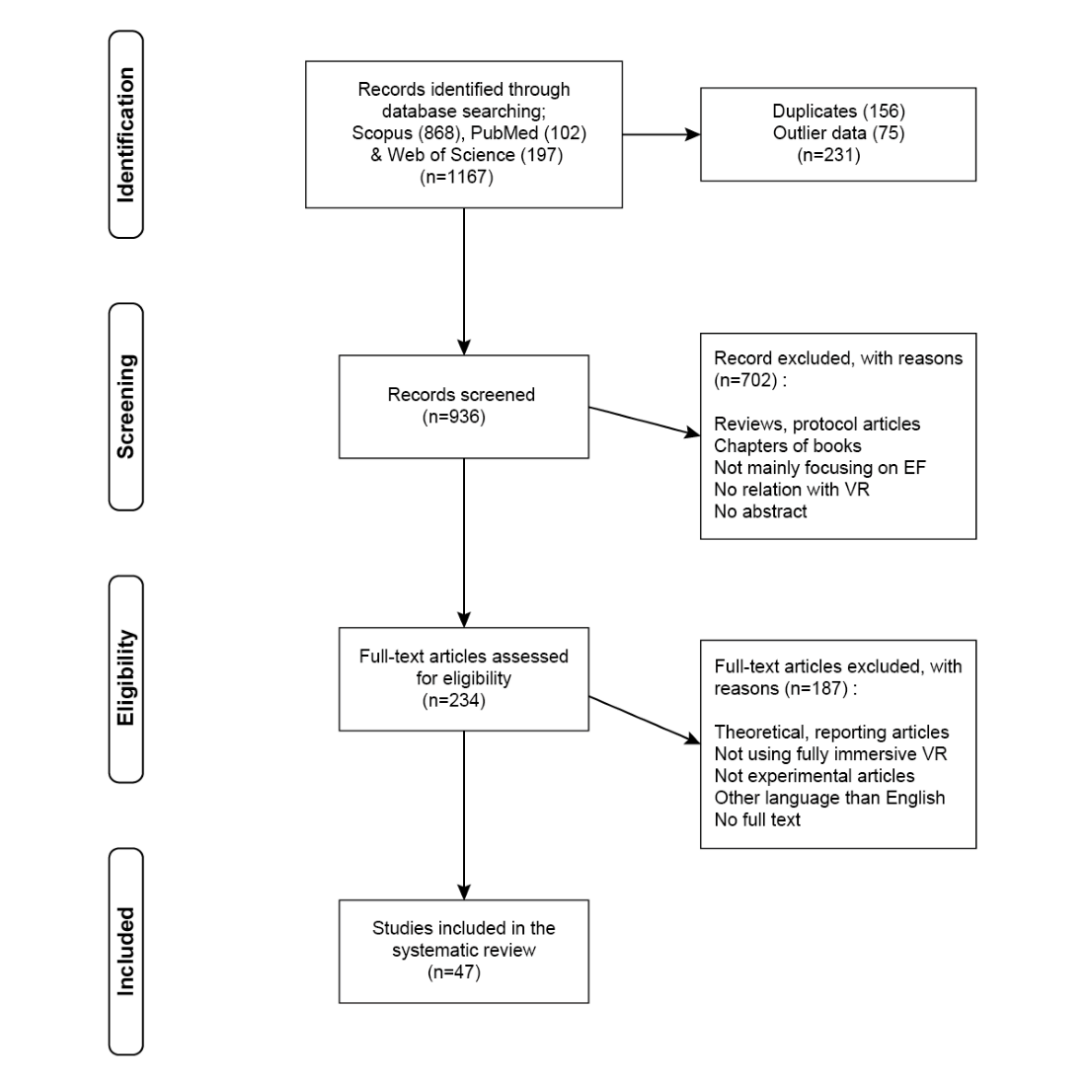
Flowchart of the process of selecting studies. EF, executive function; VR, virtual reality.

The overall procedure of the study and the number of selected articles are shown in Figure 1. After searching the web database, 1167 articles were collected (Scopus 868, PubMed 102, and Web of Science 197). After removing duplicates and outliers, the remaining articles were used in the next phase. As a result of the screening phase, 702 articles were excluded by titles, keywords, and abstract review. With abstracts of 234 papers (data set 1), keyword analysis was conducted to examine the research trends. Then, we conducted a full-text review in the eligibility phase, and 187 papers were excluded based on the exclusion criteria. Finally, 47 papers (data set 2) were selected for the descriptive analysis.

### Data Analysis

Data analysis was conducted by two sub-analysis methods.

First, a keyword analysis was conducted with data set 1. It was important to specify keywords for the analysis, so we conducted keyword extraction with Python NLTK. The Natural Language Toolkit (NLTK) is a Python package that has been developed for natural language processing and document analysis. The NLTK extracted top 200 keywords based on their frequency from each article’s title, keywords, and abstract. Among the results of the extraction, we selected significant keywords in line with the study’s purpose and categorized them according to measuring targets, names of mental disorders, and behavioral and physiological measures. Moreover, some keywords were added from the 47 full-text descriptive analysis.

Second, a descriptive analysis was conducted in detail with data set 2. The analysis was conducted with almost the same categories as the keyword analysis, but we also added some other elements: purpose of article, measuring targets, age of participants, names of mental disorders, real-time walking, and environment of VR.

All the analyses were conducted within a certain timescale. Considering the evolution of yearly publication frequencies, the time periods were divided into 4 stages of 5-year blocks: period 1, 2000-2004; period 2, 2005-2009; period 3, 2010-2014; and period 4, 2015-2019.

## Results

### Evolution of the Publication Frequency

**Figure 2:**
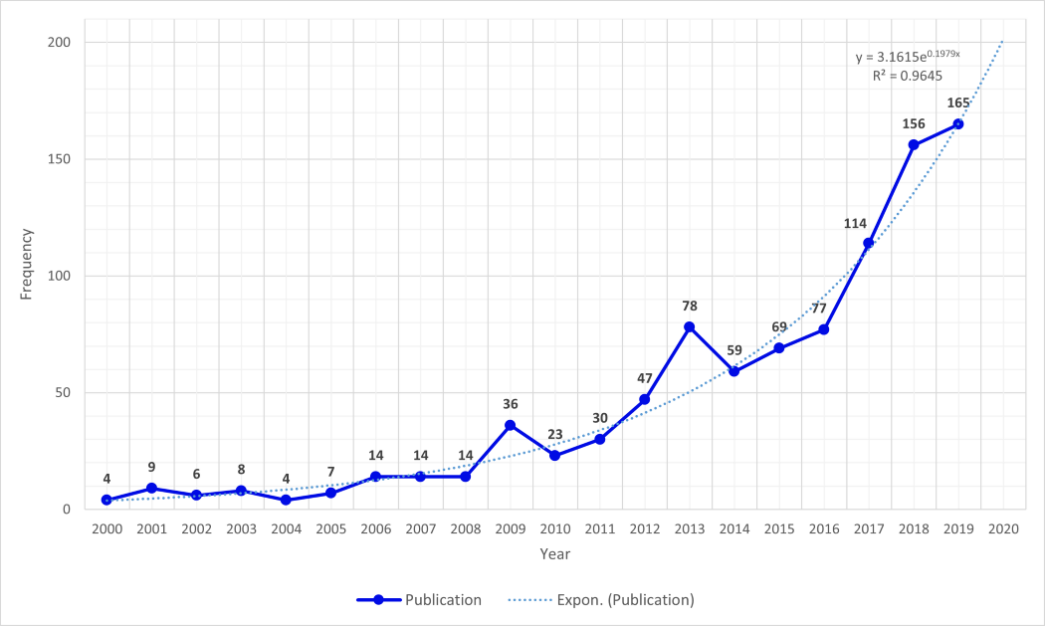
The evolution of publication frequency.

In this section, we give an overview of the study of virtual neuropsychological tests in connection with EFs. There was a change of pace of the publication frequency from 2000 to 2019, with an exponential curve, as shown in Figure 2, with 936 articles after removing duplicates and outliers. Studies maintained a certain level all through the 2000s. The publication frequency began to increase conspicuously in 2009 and has rapidly increased in the past 10 years. It expresses the high interest in VR for dealing with mental disorders.

### Keyword Analysis

A keyword analysis was applied to data set 1 that mainly focused on EFs. For the keyword analysis, we segmented the total period (2000-2019) into four periods: 2000-2004, 2005-2009, 2010-2014, and 2015-2019. A significant increase in the publications in Figure 2 was the main rationale to classify the periods. In the keyword analysis, we examined the trends in VR applying to neuropsychology.

#### Target Cognitive Abilities

**Figure 3:**
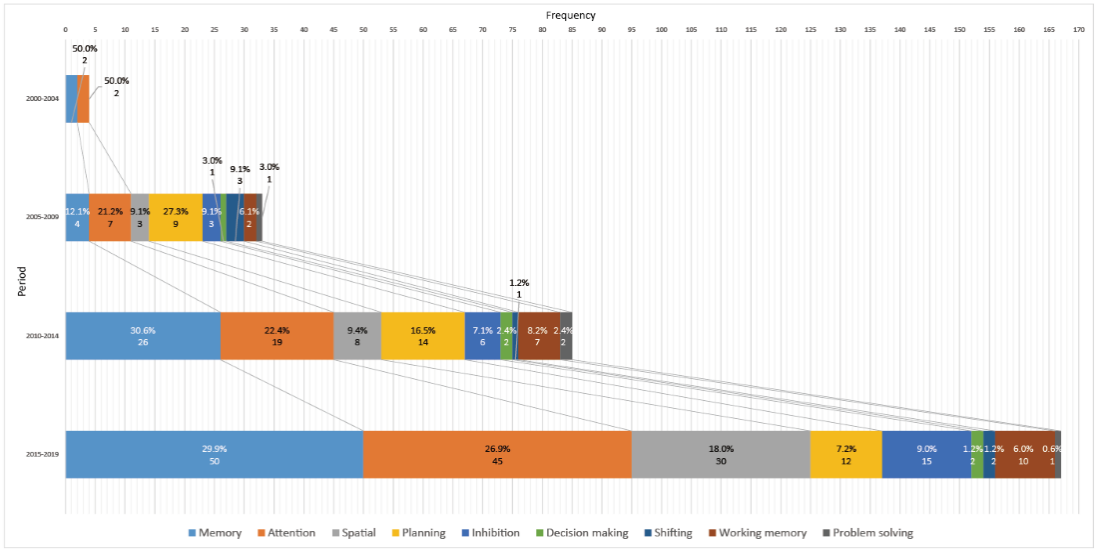
The evolution of cognitive abilities targeted in the 234 screened studies.

Cognitive ability includes various information-handling processes that occur in the human brain [39]. Target EFs brought from the theoretical background were complemented with “memory,” “attention,” and “spatial” since these frequently appeared with keyword extraction. Memory and attention are wide and basic concepts when assessing cognitive abilities [39,40]. They had a high proportion through all periods (Figure 3) because they occurred when measuring specific cognitive abilities together.

In the early 2000s, period 1, all studies tried measuring basic abilities, memory, and attention, but researchers began to try to measure other targets, including higher-order EFs. It seems that the diversification accelerated in line with the popularization of VR in the late 2000s.

#### Target Symptoms

**Figure 4:**
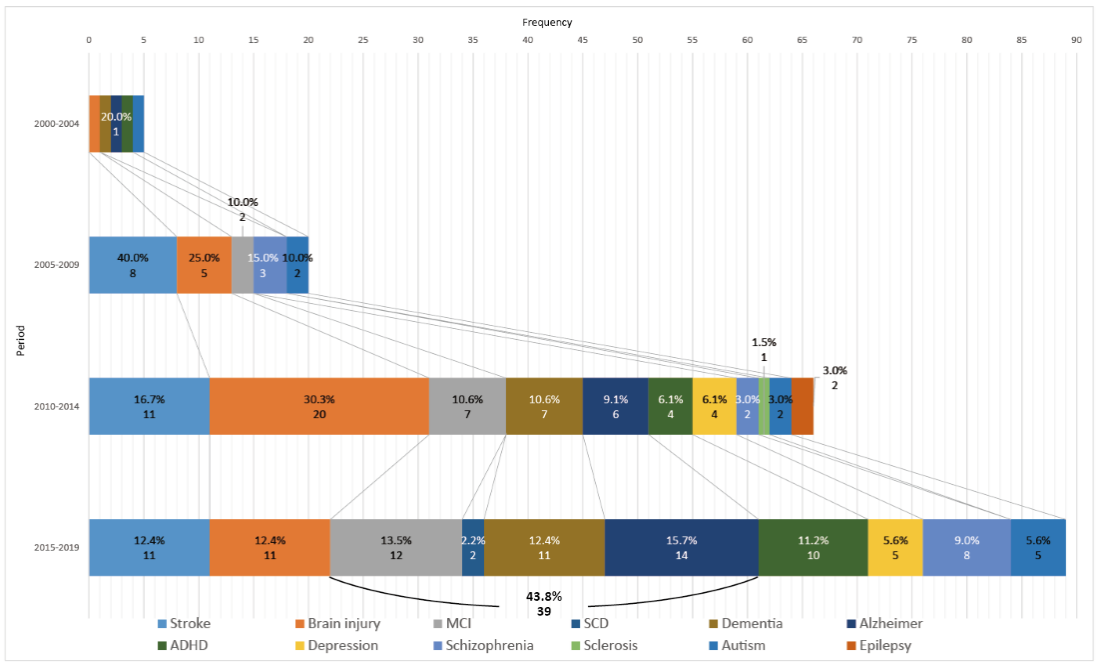
The evolution of symptoms targeted in the 234 screened studies. AD: Alzheimer’s disease; ADHD, attention deficit hyperactivity disorder; MCI: mild cognitive impairment; SCD: subjective cognitive decline.

Our mental disorder list was formed by the result of keyword extraction, and we manually added several mental disorders from the 47 articles we reviewed for full-text descriptive analysis. There was a transition stage from the late 1990s to the early 2000s, when traditional pen-and-paper tests turned into computerized tests [41]. As shown in Figure 4, only a few studies conducted experimental attempts to apply VR to neuropsychology. Period 1 was the beginning stage of using VR technology for neuropsychological assessment, and this was ongoing through period 2. After that, from the early 2010s, studies expanded to treat various types of mental disorders. Until period 3, brain injury (stroke is included in brain injury in a broad view) was the most common mental disorder in this research area, maybe due to its high incidence and fatal long-term consequences [42]. However, in period 4, studies gradually spread to other brain disorders.

Through period 4, research on the topics mild cognitive impairment (MCI), subjective cognitive decline (SCD), dementia, Alzheimer’s disease (AD), attention deficit hyperactivity disorder (ADHD), and schizophrenia increased. Among them, MCI, SCD, AD, and dementia are age-related symptoms that increased from period 3 (36.4%) to period 4 (43.8%). In this article, we view SCD, MCI, AD, and dementia as one continuous process and call it “age-related cognitive decline.” In addition, studies on ADHD increased from period 3 (6.1%) to period 4 (11.2%), and studies on schizophrenia increased from 3.0% to 9.0%. Despite a high prevalence compared with schizophrenia, autism, ADHD, and depression remained less investigated.

#### Use of Behavioral and Physiological Data

**Figure 5:**
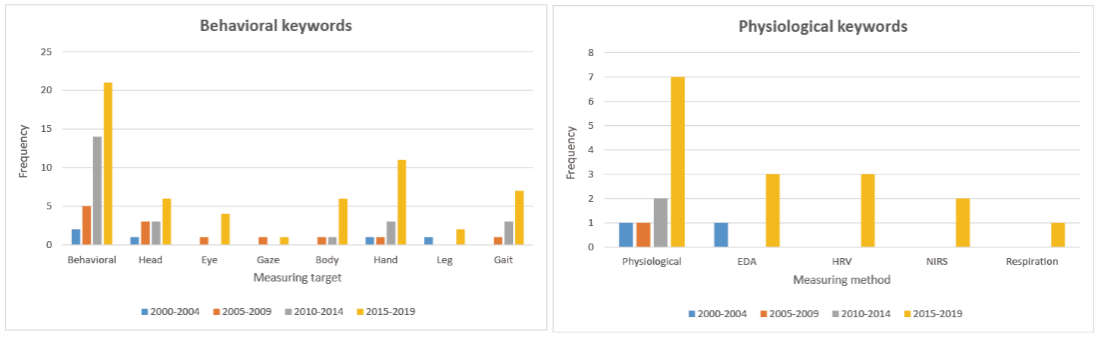
(Left) The use of behavioral data over each period. (Right) The use of physiological data over each period. EDA: electrodermal activity; HRV: heart rate variability; NIRS: near-infrared spectroscopy.

Attempts to obtain a subject’s behavior data continuously increased from the early 2000s, while attempts to obtain physiological data rapidly increased in period 4 (Figure 5). In terms of content, there were attempts to use head tracking, gaze (eye) tracking, body tracking, hand tracking, and gait (leg) tracking, as shown in Figure 5. In the graph for physiological data, there were studies completed in EDA, HRV, NIRS, and respiration measurement, but EDA was the only attempt before period 4, and the others were conducted in period 4.

### Descriptive Analysis

The final 47 articles included through PRISMA were experimental studies that conducted neuropsychological tests using fully immersive VR. The article set was divided into the same four periods as the keyword analysis. Studies on fully immersive VR were encouraged in the early 2010s, and most of the research was done within the past 5 years (period 4) with every year. Table 1 represents a summary of the 47 studies.

**Table 1:** Summary of the characteristics of the 47 studies.

Study	Journal/conference	Purpose of study	Target cognitive functions	Subject status	VE^a^	BE^b^
Areces et al (2018) [43]	*PLoS ONE*	As^c^ Val.S^d^	Attention Inhibition	ADHD^e^ Age 6-16 years	Classroom	—
Bailey et al (2019) [44]	*Journal of Applied Developmental Psychology*	Un^f^	Inhibition	Normal only Age 4-6 years	Other(s)	—
Blume et al (2017) [45]	*Trials*	Tr^g^ Val.U^h^	Attention Inhibition Working memory	ADHD Age 6-10 years	Classroom	O^i^
Chicchi et al (2019) [46]	*Applied Neuropsychology: Adult*	Val.U	Attention Decision making Inhibition Planning Shifting	Normal only Mean age 30.14 years	Other(s)	—
Chicchi et al (2019) [47]	*Frontiers in Psychology*	Un	Attention Planning Shifting	Normal only Age 18-48 years	Kitchen	O
Climent et al (2019) [48]	*Applied Neuropsychology: Adult*	Un	Attention Working memory	Normative study Age 16-90 years	Aquarium	—
Coleman et al (2019) [49]	*Frontiers in Psychology*	Tr	Attention Working memory	ADHD Age 6-13 years	Classroom	—
Dahdah et al (2017) [50]	*Neurorehabilitation*	Tr Val.U	Inhibition	Stroke, TBI^j^ Mean age 40.3 years	Apartment Classroom	—
Davison et al (2018) [51]	*Acta Neuropsychiatrica*	Val.S Val.U	Planning Working memory Motor	Normal only Age 19-24, 66-77 years	Parking lot Laboratory	—
De Lillo et al (2014) [52]	*American Journal of Primatology*	Un	Working memory	Normal only Age 18-36 years	Other(s)	—
De Luca et al (2020) [42]	*Applied Neuropsychology: Child*	Tr	Inhibition Planning Problem solving Updating Working memory Motor Spatial	TBI Age 15 years	A number of VEs	O
Díaz-Orueta et al (2014) [53]	*Child Neuropsychology*	As Val.U	Attention Inhibition	ADHD Age 6-16 years	Classroom	—
Eom et al (2019) [54]	*Cyberpsychology, Behavior and Social Networking*	As Un	Attention Inhibition	ADHD Age 6-17 years	Classroom	—
Grewe et al (2013) [55]	*Journal of NeuroEngineering and Rehabilitation*	Val.S	Working memory Spatial	Epilepsy Age 25-47 years	Market	—
Grewe et al (2014) [56]	*Epilepsy and Behavior*	As Val.S	Working memory Spatial	Epilepsy Age>18 years	Market	—
Henry et al (2012) [57]	*Journal of Neuroscience* *Methods*	Val.U	Attention Inhibition Motor	Normal only Age 19-58 years	Apartment	—
Huang (2020) [58]	*Cyberpsychology Behavior and Social Networking*	Tr Un	Inhibition Shifting Working memory	Normal only Age>50 years	Other(s)	—
Ijaz et al (2019) [59]	*Journal of Medical Internet Research*	As Val.S	Working memory Spatial	Normal only Mean age 73.22 years	Google Street View	O
Iriarte et al (2012) [60]	*Journal of Attention Disorder*	Un	Attention Inhibition	Normative study Age 6-16 years	Classroom	—
Kang et al (2008) [61]	*Cyberpsychology and Behavior*	As	Attention Planning Working memory	Stroke Mean age 55.4 years	Market	—
Lalonde et al (2013) [29]	*Journal of Neuroscience* *Methods*	Val.U	Inhibition	Normal only Age 13-17 years	Classroom	—
Liao et al (2019) [62]	*Frontiers in Aging Neuroscience*	Tr Val.U	Attention Inhibition Planning Shifting Working memory Motor	MCI^k^ Age>65 years	A number of VEs	O
Lo Priore et al (2003) [63]	*Cyberpsychology and Behavior*	Un	Attention Planning Shifting	Normal only Young age	Market	O
Mondellini et al (2018) [64]	*2018 IEEE 6th International Conference on* *Serious Games and Appli* *cations for Health, SeGAH 2018*	Tr	Working memory	Normal only Age<40 years	Market	—
Negut et al (2017) [65]	*Child Neuropsychology*	As Val.S Val.U	Attention Inhibition	ADHD Age 7-13 years	Classroom	—
Nolin et al (2009) [66]	*Annual Review of CyberTherapy and Telemedicine*	Val.U	Attention Inhibition	TBI Age 8-12 years	Classroom	—
Nolin et al (2016) [67]	*Computers in Human Behavior*	Val.S Un	Attention Inhibition	Normal only Age 7-16 years	Classroom	—
Ouellet et al (2018) [68]	*Journal of Neuroscience* *Methods*	Val.S Un	Working memory	SCD^l^ Mean age 67.2 years	Market	—
Parsons et al (2013) [69]	*Journal of Clinical and* *Experimental Neuropsy* *chology*	Val.U	Attention Inhibition	Normal only Age 18-28 years	In a car	O
Parsons et al (2016) [70]	*Journal of Autism and Developmental Disorder*	As Val.U	Inhibition	Autism Age 18-34 years	Classroom	—
Parsons et al (2017) [71]	*Journal of Neuroscience* *Methods*	As Val.S	Working memory	Normal only Age>17 years	Market	—
Parsons et al (2018) [72]	*IEEE Transactions on Affective Computing*	As	Attention Inhibition	Normal only Age 18-28 years	In a car	O
Parsons et al (2018) [73]	*Journal of Neuroscience* *Methods*	Val.U	Inhibition	Normal only Age 18-30 years	Apartment	—
Parsons et al (2019) [74]	*Applied Neuropsychology: Adult*	As	Attention Inhibition	Normal only Older adults and undergraduate young adults	Apartment	—
Plechatá et al (2019) [75]	*Frontiers in Psychology*	As Un	Decision making Shifting Working memory	Normal only Age 19-39, 60-91 years	Market	—
Plotnik et al (2017) [76]	*International Conference on Virtual Rehabilitation (ICVR)*	Val.S	Attention Planning	Normal only Mean age 37.1 years	Other(s)	O
Proulx et al (2018) [77]	*Annual Review of CyberTherapy and* *Teleme* *dicine*	Un	Attention Inhibition Working memory	Normal only Age 20-60 years	Laboratory Tent	—
Rizzo et al (2000) [78]	*Cyberpsychology and Behavior*	As	Attention Inhibition	ADHD Age 8-12 years	Classroom	—
Robitaille et al (2017) [79]	*Disability and Rehabili* *tation: Assistive Technology*	As	Attention Motor	TBI Mean age 30.3 years	Middle-east Village	O
Serino et al (2018) [80]	*Sensors (Switzerland)*	As	Working memory Spatial	AD^m^ Age>65 years	Other(s)	—
Tarnanas et al (2013) [81]	*International Conference on* *Virtual Re* *habilitation,* *ICVR* *2013*	As Un	Inhibition Planning Working memory Motor	MCI Age >60 years	Apartment	O
Tarnanas et al (2013) [82]	*Journal of Medical Internet Research Serious Game*	As Val.S	Inhibition Shifting Updating	MCI, AD Age>65 years	Apartment	O
Tarnanas et al (2014) [83]	*Alzheimer’s and Dementia*	As	Inhibition Shifting Updating	MCI Mean age 71.6 years	Apartment	O
Voinescu et al (2019) [84]	*26th* *IEEE Conference on Virtual Reality and 3D User Interfaces, VR 2019 – Proceeding*	Val.S Val.U	Attention Inhibition	Normal only Age 23-51 years	Aquarium	—
Yasuda et al (2017) [85]	*Topics in Stroke Rehabilitation*	Tr Val.S	Spatial	Stroke Age 45-85 years	Other(s)	—
Yeh et al (2012) [86]	*2012 IEEE-EMBS Conference on Biomedical* *Engineering and Scien* *ces, IECBES 2012*	As	Attention Decision making Shifting Working memory	ADHD Age 7-13 years	Classroom	O
Yeh et al (2012) [87]	*2012 IEEE-EMBS Conference on Biomedical* *Engineering and Scien* *ces, IECBES 2012*	As	Planning Working memory	Dementia Age 60-90 years	Market	O

^a^VE: virtual environment.

^b^BE: behavior or ecological measures.

^c^As: assessment.

^d^Val.S: structure validation.

^e^ADHD: attention deficit hyperactivity disorder.

^f^Un: uncategorized.

^g^Tr: treatment.

^h^Val.U: usability validation.

^i^O: includes BE measures.

^j^TBI: traumatic brain injury.

^k^MCI: mild cognitive impairment.

^l^SCD: subjective cognitive decline.

^m^AD: Alzheimer’s disease.

#### Analysis of Purpose of the Studies

This study identified that the purpose of the 47 articles is mainly clustered into four groups, and some additional purposes also existed. Thus, in this study, we divided the articles into five groups: assessment, treatment, usability validation, structure validation, and, for additional purposes, uncategorized: (1) assessment: to assess, diagnose, or evaluate a neuropsychological condition of the subject; (2) treatment: to treat or rehabilitate the patient; (3) usability validation: to validate the efficiency of a VR application by comparing it with an existing traditional neuropsychological test; (4) structure validation: to attempt to test the construct integrity and ecological and temporal stability of the VR itself; and (5) uncategorized: to evaluate presence, cybersickness, aging, and normative studies. Since there could be multiple purposes in one article, we checked all articles for all purposes.

**Figure 6:**
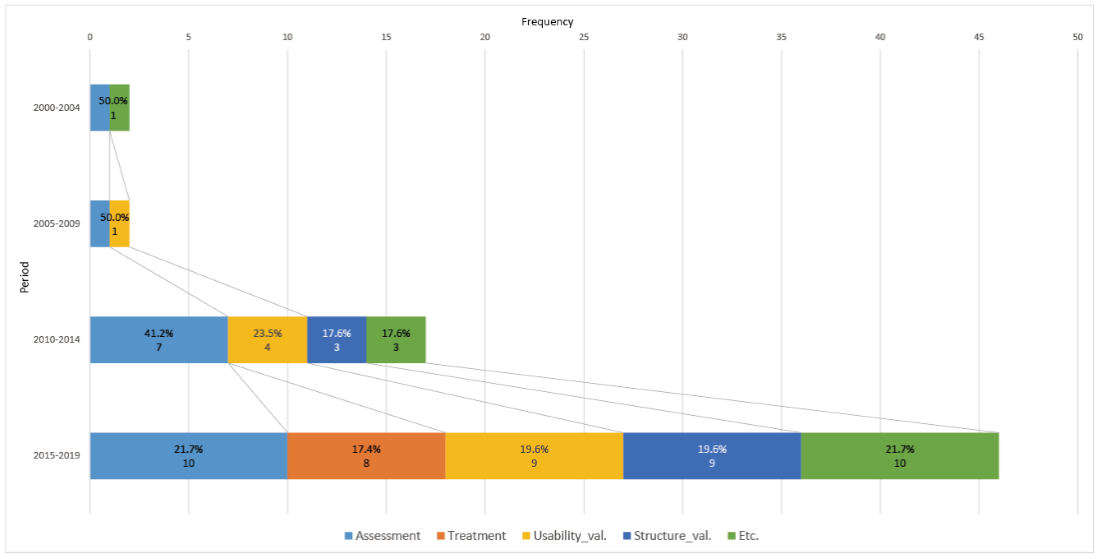
The evolution of the objective of the final 47 studies.

As shown in Figure 6, the majority of the studies were conducted for assessment and validation. The number of validation studies was nearly steady through all periods. The number of studies dramatically increased as we moved to period 3. In period 3, 7 studies (41.2%) reported the results of assessing EF(s), 7 studies (41.2%) tried to validate their VR neuropsychological tests, but there were no studies for applying a VR test to treatment until period 4. Treatment studies first appeared in 2017, and the tendency began to expand. In period 4, 10 (21.7%), 8 (17.4%), and 18 (39.2%) studies were conducted on assessment, treatment, and validation, respectively.

Meanwhile, the proportion of uncategorized studies increased rapidly in period 4. Before period 4, there were just 4 studies, but 10 studies corresponded to the uncategorized type. Of the four studies, one was a study for the sense of presence that was conducted early in VR history, and the others were conducted in period 3 to obtain information about certain cognitive functions associated with functional impairment in ADL. However, the uncategorized studies in period 4 were slightly different: 7 (70%) of the 10 studies were conducted to evaluate the sense of presence and cybersickness of users in the VE, and the remainder were studies on age-specific differences.

#### Cognitive Abilities and VEs

The VEs used to target EF(s) are shown in Table 2. In addition to the cognitive abilities selected in the Target Cognitive Abilities section, we added spatial (navigation, spatial perception, etc) and motor (psychomotor, motion, movement, etc) abilities, because these are significant features within fully immersive VR tests. In general, most articles not only target a certain cognitive ability but also treat several abilities, so we allowed for multiple checking.

**Table 2:** Frequency of VEs^a^ by EFs^b^.

Cognitive ability	VEs	Reference	Number of papers that studied the specific cognitive ability (n)
Inhibition (n=27)	Apartment	[50,57,73,74,81-83]	7
	Classroom	[29,43,45,50,53,54,60,65-67,70,78]	12
	Aquarium	[84]	1
	Laboratory	[77]	1
	Tent outdoors	[77]	1
	In a car	[69,72]	2
	Others^c^	[42,44,46,58,62]	5
Working memory (n=21)	Laboratory	[51,77]	2
	Parking lot	[51]	1
	Market	[55,56,61,64,68,71,75,87]	8
	Classroom	[45,49,86]	3
	Tent outdoors	[77]	1
	Google Street View	[59]	1
	Aquarium	[48]	1
	Others	[42,52,58,62,80]	5
Shifting (n=9)	Apartment	[82,83]	2
	Market	[63,75]	2
	Classroom	[86]	1
	Kitchen	[47]	1
	Others	[46,58,62]	3
Decision making (n=3)	Classroom	[86]	1
	Market	[75]	1
	Others	[46]	1
Problem solving (n=1)	Others	[42]	1
Planning (n=10)	Parking lot	[51]	1
	Laboratory	[51]	1
	Market	[61,63,87]	3
	Apartment	[81]	1
	Kitchen	[47]	1
	Others	[42,46,62,76]	4
Updating (n=3)	Apartment	[82,83]	2
	Others	[42]	1
Attention (n=24)	Classroom	[43,45,49,53,54,60,65-67,78,86]	11
	Market	[61,63]	2
	Aquarium	[48,84]	2
	In a car	[69,72]	2
	Kitchen	[47]	1
	Laboratory	[77]	1
	Tent outdoors	[77]	1
	Apartment	[57]	1
	Middle-east village	[79]	1
	Others	[46,62,76]	3
Spatial (n=6)	Market	[55,56]	2
	Google Street View	[59]	1
	Others	[42,80,85]	3
Motor (n=6)	Parking lot	[51]	1
	Laboratory	[51]	1
	Apartment	[57,81]	2
	Middle-east village	[79]	1
	Others	[42,62]	2

^a^VE: virtual environment.

^b^EF: executive function.

^c^Others: does not use specific environments, uses multiple environments as a minigame with multiple scenarios, or uses a certain unrealistic environment that could not be experienced in real life.

Since the study of fully immersive VR began, numerous VEs have been designed. In the initial stage, until 2012, only classrooms and supermarkets were studied, classrooms four times [60,66,78,86] and supermarkets three times [61,63,87], but VEs began to diversify as research progressed.

Inhibition and attention were targeted the most, as they are deeply related functionally and conceptually. They were studied 27 and 24 times, respectively. For inhibition and attention, virtual classroom environments were mostly used, and virtual apartments were the next most common type of environment. The next most targeted EF was working memory, which was studied 21 times. For working memory, various VEs were used, but markets were the most common, with eight studies. Next, planning was studied 10 times, shifting was studied 9 times, spatial and motor were examined 6 times each, and decision making, updating, and problem solving were studied 3, 2, and 1 times, respectively.

#### Description of Participants

**Figure 7:**
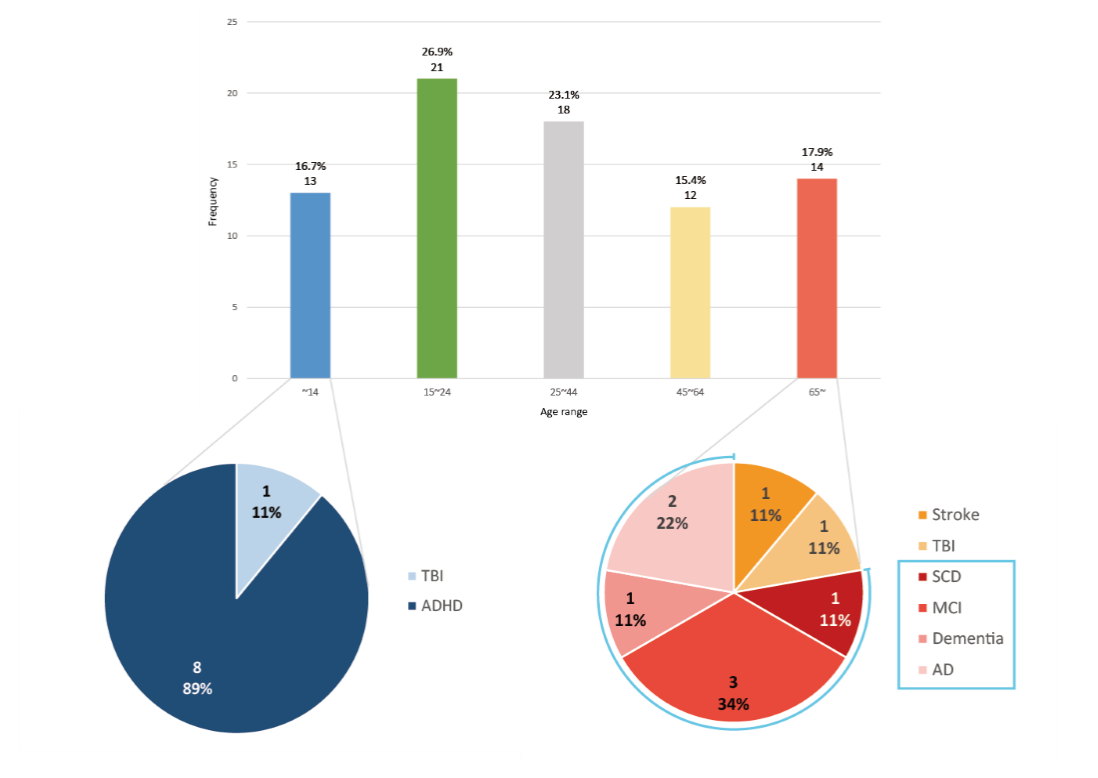
Research based on the age of the participant and symptoms of two age groups, under 14 and over 65 years. AD: Alzheimer’s disease; ADHD: attention deficit hyperactivity disorder; MCI: mild cognitive impairment; SCD: subjective cognitive decline; TBI: traumatic brain injury.

An overall distribution of the participants’ age is presented in Figure 7. For age sorting, the United Nations’ age classification guideline for health, health services, and nutrition was applied [88]. Some articles that did not perfectly fit the guideline were checked multiple times according to their range of participants.

The age distribution of participants in all the articles was similar in all periods. The proportion of 15-24-year-olds was the highest, and the proportion of those 65 years and was the third. In the under-14 age group, there is just one article that studied traumatic brain injury (TBI), and the others examined ADHD. Among those above 65 years, the age-related cognitive decline corresponded to nearly 80% of all articles. In addition, there were many studies with only a healthy group.

#### The Emphasis on Real-Time Walking

The use of a multidirectional treadmill to allow a subject the possibility of walking by themselves first appeared in period 3. Research using treadmills usually targeted patients with TBI and MCI to rehabilitate their gate and motor ability. Entering period 4, some VR applications began to provide real-time walking without any equipment. In fully immersive VR, supermarkets, kitchens, laboratories, and small squares were used as VEs, but supermarkets corresponded to half of all environments.

Fully immersive VR provides a sense of reality and of presence in virtual spaces with a special apparatus, such as an HMD [21]. Furthermore, another merit of fully immersive VR is that it has a high level of ecological validity by allowing free movement in a certain space. The real-time walking studies can be separated into two categories, real-time walking and walking on a treadmill. In the case of a treadmill, fully immersive environments are built with a 180°/360° or a dome-shaped screen [42,76,83]. For real-time walking, VR can function as a tool allowing ADL.

## Discussion

### Expansion of Research: From Assessment to Treatment and From Simple Tasks to ADL

The most noticeable phenomenon is the appearance of treatment studies in period 4. In the early periods, when VR was first used in neuropsychological tests, it was only used for assessing cognitive states. However, high ecological validity, reproducibility of daily life, and the possibility of measuring behavior responses that were not available with traditional neuropsychological tests opened a new venue for cognitive rehabilitation [42,64,89,90]. Therefore, the research trend changed from being assessment oriented toward an additional interest for treatments. The possibility of treatment of cognitive problems using VR seems to expand continuously. In these studies, researchers have tried to train or rehabilitate the participants’ cognitive abilities (mainly inhibition and working memory) through regular and repetitive VR tasks that consisted of daily-living scenarios or game-like applications [42,58,62,64]. Some research argues that the biomarker and the degree of brain activation obtained as kinematic outcomes are indicators of treatment response [91,92].

From the perspective of changes in validation, it seems that there are two main streams. The first perspective is about transplanting existing traditional tests into VR, such as the Stroop test [29,50,57,67,69,70,73] and the trail-making task [76]. This includes most of the usability validation studies in data set 2. Because the Stroop test is a simple and ocular task, it has been considered suitable to convert into a VE. In addition, there is one study that brings the trail-making test into the VE. Second, an interesting thing is that most of the structure validation studies were conducted in supermarket environments based on the multiple-errand test [55,56,68,71]. Supermarket environments are popular due to their deep relation with daily living. Researchers have also tried to design various scenarios that are related to daily living, such as parking simulation [51] and pathfinding with Google Street View [59]. However, continuing to adapt parameters or structural properties to suit VR neuropsychological tests remains a challenge.

The increase in uncategorized studies in period 4 could also be seen in the same context. Most of the uncategorized studies examined the sense of presence and cybersickness [44,47,54,58,63,67,75,77]. These are indeed the two major problems for wider acceptance of VR for more age groups and for obtaining more ecological validity. The increase in studies to solve these problems aims for participants to feel more comfortable, which could further lead to the development of daily-living contents and expand the area of treatment. Therefore, solving these problems seems a key point to improve fully immersive neuropsychological testing in VR, but there is still a long way to go.

### Diversification of Targeted EFs and VEs

As shown in Figure 3, diversification started in line with the popularization of VR in the late 2000s, and the research has grown quantitatively and qualitatively ever since. Initially, studies mostly targeted low-order EFs, such as inhibition, attention, and working memory [61,66,78]. However, in recent years, VR technology has evolved to overcome the problem of presence and cybersickness. This enabled researchers to implement higher ecological ADL with various practical scenarios and VEs. Consequently, it has led to studies that deal with higher-order EFs, such as decision making, planning, and problem solving.

Inhibition and attention are functions deeply related to ADHD. Because most subjects with ADHD are children and youth, it seems that researchers have actively used classroom and apartment (more accurately, the living room) environments that those ages are familiar with [43,45,53,54,65,78]. As the Stroop test is famous for assessing one’s inhibition, many studies on inhibition and attention have translated it into VR. Especially, most studies conducted under virtual apartment environments have developed their application based on the Stroop test [50,57,73,74]. Since familiarity with the participants’ daily-living situation induces psychological comfortability, application can sophisticatedly measure the natural state of cognitive abilities.

The supermarket environment is most frequently used for measuring working memory. Planning [61,63,87] and decision making [75] are also associated with the supermarket environment. For higher-order EFs, various other environments and daily-living scenarios have been tried, such as laboratories [51,77], kitchens [47], and parking simulation [51]. It is unrealistic to precisely measure one isolated EF due to its interdependent property. Higher-order EFs should be comprehensively measured. Only one case measured planning alone, a study that was the first attempt to apply the trail-making test to immersive VR [76]. Lastly, studies on spatial and motor abilities were tried after 2012 using a treadmill or motion-tracking systems.

Since one of the EFs closely connected to other EFs we defined, EFs in ADL tend to be treated comprehensively rather than alone. These deal with specific situations, the emergence of distractors, and well-organized scenarios that assist the original task.

### Diversification of Target Symptoms and Expansion of the Age Group

As shown in Figure 4, target symptoms began to diversify after period 2. Stroke and brain injury accounted for the largest percentage until period 3, but in period 4, this changed. This indicates that VR studies on neuropsychology are being conducted in a wide variety of conditions. The thing to keep an eye on is the conspicuous increase of age-related cognitive decline and a relatively high proportion of ADHD.

The many VR studies have focused on ADHD, which is a popular symptom of young age in the early stage of VR research. In Figure 7, the under-14 age group is composed of TBI and ADHD, and ADHD represents the majority of these studies. It is not surprising as almost 5% of children are diagnosed with ADHD [86]. Many previous studies have reported the positive effect of VR on children who have a mental disorder [93-95] and surely on ADHD [43,78,86]. In addition, the ease of converting the Stroop test into immersive VR would have played a role, since the Stroop test is a neuropsychological assessment to measure attention and inhibition, which are major deficit features of ADHD [43]. If we regard under 25 years as one unique group, the activeness of related research can be seen as familiarity and accessibility to VR and resistance to cybersickness of adolescents. Young people are more adaptable to VR, even immersive VR [96].

As VR technology advances and related research accumulates, obstacles that blocked the application of VR neuropsychological tests to the elderly are being resolved [97]. Figure 7 also shows the expansion of the research to the elderly. In the 65+ age group, about 80% of papers are related to age-related cognitive decline. As mentioned before, we consider SCD, MCI, AD, and dementia together as age-related cognitive decline. MCI is a symptom that decreases one’s memory, attention, and cognitive function, and it could lead to dementia or AD [98]. SCD is an early state when someone’s cognitive ability is beginning to decline [99]. In other words, SCD may indicate a pre-MCI condition, even an early marker for the symptomatic manifestation of dementia. Age-related cognitive decline symptoms are common in the elderly. In addition, depression is a quite under-investigated area despite a huge impact on daily-living executive dysfunction in 60% of depressive states in the elderly, with an exponential growth of prevalence with age, a poorer response to antidepressants, and more evolution toward dementia compared to depression without dysexecutive syndrome [100-102].

Investigating VR as a neuropsychological tool for the elderly seems increasingly realistic since VR has become more familiar even for seniors. As we have seen before, there has been a lot of research on and technological advancement in presence and cybersickness in VR, and it is possible that the public and even the elderly could comfortably access it [103-105], which enables expansion to the elderly and causes rapid growth of the research that targets age-related cognitive decline symptoms after period 4.

Overall, immersive VR offers a high number of possibilities to deal with neuropsychological symptoms in various age groups. It shows the versatility of VR, which is applicable from youth with ADHD to the elderly with age-related cognitive decline.

### Combining Behavioral and Physiological Data Measurement

It seems that measuring behavioral data and physiological data is becoming increasingly important. These kinds of data are used to extend the concept of embodied cognition to measure the neuropsychological status and to overcome the weaknesses of VR.

Embodied cognition is a broad notion from embodiment thesis in philosophy, and it states that an agent’s cognition is powerfully affected by aspects of their body beyond the brain itself. This means that sensorimotor experiences and actions are crucial to cognitive processing [106]. These aspects are thought to be crucial for cognitive performances in the elderly [107]. In this context, measuring behavioral and physiological variables in immersive VR would be a key method of assessing the neuropsychological status. Thus, the growth of associated research seems to be increasing because of the ecological characteristics of VR environments. In addition, when we looked deeper into gait or walking, there was a large difference in the appearance of walking on a treadmill and real-time walking in periods 3 and 4. With real walking in a VR neuropsychological test, researchers were able to apply the concept of embodied cognition and measure it in ADL.

Furthermore, an increase in physiological data measurements should be considered as these can provide auxiliary ways to assess the neuropsychological status of a patient. In addition, it is also considered to be a helpful way to overcome cybersickness [108-110]. Cybersickness is a major obstacle in applying VR to older people and people who have difficulties adapting to VR because they feel dizzy. In addition, research on emotion recognition and presence in VR uses physiological data [111-114]. With these efforts added, VR neuropsychological tests’ versatility for all ages continues to improve.

Overall, by using behavioral and physiological data measurement, VR neuropsychological tests are not only getting over existing drawbacks but also expanding to the concept of embodied cognition to improve its measurement capability.

### Limitations

This review had a few limitations. First, keyword analysis was conducted by only using titles and abstracts of the articles. This means that there is a possibility of a difference with the articles’ main texts. Second, we only included articles that described research conducted with real participants, so conceptual and theoretical trends in the area were not covered. Finally, not every article markedly described the target EFs. Likewise, there were some ambiguities in clearly assigning the purpose of each article to assessment, treatment, structure validation, or usability validation. Even though we attempted to judge based on the description of what EFs the articles targeted, and what their intended purpose was, there was still room for error. Future research could include broader studies in the area and use more rigorous methods to analyze the trends.

### Conclusion

This review will assist researchers in understanding the trends in VR neuropsychological tests over the past 20 years. Associated research has been on the rise and has sharply increased in recent years. In this process, the advancements in technology and various approaches have led to diversified target cognitive abilities, including EFs, as well as target symptoms. Moreover, collecting behavioral and physiological data enables a wide understanding about treating EFs by using the concept of embodied cognition. As a result, VR neuropsychological tests now cover a wide range of age groups and extend beyond assessment tools to treatment tools. This review shows that there is a continuously increased interest in dealing with neuropsychology by using fully immersive VR, and it is expected that this will help advance research in this area.

## Abbreviations

AD: Alzheimer’s disease
ADHD: attention deficit hyperactivity disorder
ADL: activities of daily living
BE: behavior or ecological measures
CAVE: Cave Automatic Virtual Environment
EDA: electrodermal activity
EF: executive function
HMD: head-mounted display
HRV: heart rate variability
IADL: instrumental activities of daily living
MCI: mild cognitive impairment
NIRS: near-infrared spectroscopy
NLTK: Natural Language Toolkit
PRISMA: Preferred Reporting Items for Systematic Reviews and Meta-Analyses
SCD: subjective cognitive decline
TBI: traumatic brain injury
VE: virtual environment
VR: virtual reality

## References

[1] Botella C, Fernández-Álvarez Javier, Guillén Verónica, García-Palacios Azucena, Baños Rosa (2017). Recent progress in virtual reality exposure therapy for phobias: a systematic review. Curr Psychiatry Rep.

[2] Milgram P, Kishino F (1994). A taxonomy of mixed reality visual displays. IEICE Trans Inf Syst.

[3] Ott M, Freina L (2015). A Literature Review on Immersive Virtual Reality in Education: State Of The Art and Perspectives.

[4] Mujber T, Szecsi T, Hashmi M (2004). Virtual reality applications in manufacturing process simulation. J Mater Process Technol.

[5] Waltemate T, Gall D, Roth D, Botsch M, Latoschik ME (2018). The impact of avatar personalization and immersion on virtual body ownership, presence, and emotional response. IEEE Trans Visual Comput Graphics.

[6] Gutiérrez F, Pierce J, Vergara V, Coulter R, Saland L, Caudell T, Goldsmith T, Alverson D (2007). The effect of degree of immersion upon learning performance in virtual reality simulations for medical education. Stud Health Technol Inform.

[7] Kim O, Pang Y, Kim J (2019). The effectiveness of virtual reality for people with mild cognitive impairment or dementia: a meta-analysis. BMC Psychiatry.

[8] Lezak M (2012). Neuropsychological Assessment. 5th ed.

[9] Valladares-Rodríguez S, Pérez-Rodríguez R, Anido-Rifón L, Fernández-Iglesias M (2016). Trends on the application of serious games to neuropsychological evaluation: a scoping review. J Biomed Inform.

[10] Partington J, Leiter R (1949). Partington's pathways test. Psychol Serv Center J.

[11] Stroop JR (1935). Studies of interference in serial verbal reactions. J Exp Psychol.

[12] Howieson D (2019). Current limitations of neuropsychological tests and assessment procedures. Clin Neuropsychol.

[13] Nef T, Chesham A, Schütz N, Botros AA, Vanbellingen T, Burgunder J, Müllner J, Martin Müri R, Urwyler P (2020). Development and evaluation of maze-like puzzle games to assess cognitive and motor function in aging and neurodegenerative diseases. Front Aging Neurosci.

[14] Neguț A, Matu S, Sava FA, David D (2016). Virtual reality measures in neuropsychological assessment: a meta-analytic review. Clin Neuropsychol.

[15] Alvarez JA, Emory E (2006). Executive function and the frontal lobes: a meta-analytic review. Neuropsychol Rev.

[16] Chaytor N, Schmitter-Edgecombe M (2003). The ecological validity of neuropsychological tests: a review of the literature on everyday cognitive skills. Neuropsychol Rev.

[17] Elkind JS, Rubin E, Rosenthal S, Skoff B, Prather P (2001). A simulated reality scenario compared with the computerized Wisconsin card sorting test: an analysis of preliminary results. Cyberpsychol Behav.

[18] Schultheis M, Himelstein J, Rizzo A (2002). Virtual reality and neuropsychology: upgrading the current tools. J Head Trauma Rehabil.

[19] Maggio MG, De Cola MC, Latella D, Maresca G, Finocchiaro C, La Rosa G, Cimino V, Sorbera C, Bramanti P, De Luca R, Calabrò RS (2018). What about the role of virtual reality in Parkinson disease's cognitive rehabilitation? Preliminary findings from a randomized clinical trial. J Geriatr Psychiatry Neurol.

[20] Maggio M, De Luca R, Molonia F, Porcari B, Destro M, Casella C, Salvati R, Bramanti PT, Calabro RS (2019). Cognitive rehabilitation in patients with traumatic brain injury: a narrative review on the emerging use of virtual reality. J Clin Neurosci.

[21] Valmaggia LR, Latif L, Kempton MJ, Rus-Calafell M (2016). Virtual reality in the psychological treatment for mental health problems: a systematic review of recent evidence. Psychiatry Res.

[22] Knight RG, Titov N (2012). Use of virtual reality tasks to assess prospective memory: applicability and evidence. Brain Impair.

[23] Pollak Y, Weiss P, Rizzo A, Weizer M, Shriki L, Shalev R (2009). The utility of a continuous performance test embedded in virtual reality in measuring adhd-related deficits. J Dev Behav Pediatr.

[24] Overdorp E, Kessels R, Claassen J, Oosterman J (2016). The combined effect of neuropsychological and neuropathological deficits on instrumental activities of daily living in older adults: a systematic review. Neuropsychol Rev.

[25] Tomaszewski Farias S, Cahn-Weiner DA, Harvey DJ, Reed BR, Mungas D, Kramer JH, Chui H (2009). Longitudinal changes in memory and executive functioning are associated with longitudinal change in instrumental activities of daily living in older adults. Clin Neuropsychol.

[26] Pérès K, Helmer C, Amieva H, Orgogozo J, Rouch I, Dartigues J, Barberger-Gateau P (2008). Natural history of decline in instrumental activities of daily living performance over the 10 years preceding the clinical diagnosis of dementia: a prospective population-based study. J Am Geriatr Soc.

[27] Wicklund A, Johnson N, Rademaker A, Weitner B, Weintraub S (2007). Profiles of decline in activities of daily living in non-Alzheimer dementia. Alzheimer Dis Assoc Disord.

[28] Schmeidler J, Mohs RC, Aryan M (1998). Relationship of disease severity to decline on specific cognitive and functional measures in Alzheimer disease. Alzheimer Dis Assoc Disord.

[29] Lalonde G, Henry M, Drouin-Germain A, Nolin PA, Beauchamp MH (2013). Assessment of executive function in adolescence: a comparison of traditional and virtual reality tools. J Neurosci Methods.

[30] Lezak MD (1982). The problem of assessing executive functions. Int J Psychol.

[31] Anderson P (2002). Assessment and development of executive function (EF) during childhood. Child Neuropsychol.

[32] Hughes C (2002). Executive functions and development: why the interest?. Inf Child Develop.

[33] Lehto J, Juujärvi P, Kooistra L, Pulkkinen L (2003). Dimensions of executive functioningvidence from children. Br J Dev Psychol.

[34] Miyake A, Friedman NP, Emerson MJ, Witzki AH, Howerter A, Wager TD (2000). The unity and diversity of executive functions and their contributions to complex "frontal lobe" tasks: a latent variable analysis. Cogn Psychol.

[35] Lunt L, Bramham J, Morris R, Bullock P, Selway R, Xenitidis K, David AS (2012). Prefrontal cortex dysfunction and 'jumping to conclusions': bias or deficit?. J Neuropsychol.

[36] Collins A, Koechlin E (2012). Reasoning, learning, and creativity: frontal lobe function and human decision-making. PLoS Biol.

[37] Diamond A (2013). Executive functions. Annu Rev Psychol.

[38] Liberati A, Altman DG, Tetzlaff J, Mulrow C, Gøtzsche PC, Ioannidis JPA, Clarke M, Devereaux PJ, Kleijnen J, Moher D (2009). The PRISMA statement for reporting systematic reviews and meta-analyses of studies that evaluate healthcare interventions: explanation and elaboration. BMJ.

[39] Gupta M, Musilek P (2000). Fuzzy neural networks and cognitive modeling. Int J Gen Syst.

[40] Cartwright K (2012). Insights from cognitive neuroscience: the importance of executive function for early reading development and education. Early Educ Dev.

[41] Zygouris S, Tsolaki M (2018). Handbook of Research on Innovations in the Diagnosis and Treatment of Dementia.

[42] De Luca R, Portaro S, Le Cause M, De Domenico C, Maggio M, Cristina Ferrera M, Giuffrè G, Bramanti A, Calabrò RS (2020). Cognitive rehabilitation using immersive virtual reality at young age: a case report on traumatic brain injury. Appl Neuropsychol Child.

[43] Areces D, Dockrell J, García T, González-Castro P, Rodríguez C (2018). Analysis of cognitive and attentional profiles in children with and without ADHD using an innovative virtual reality tool. PLoS One.

[44] Bailey JO, Bailenson JN, Obradović J, Aguiar NR (2019). Virtual reality's effect on children's inhibitory control, social compliance, and sharing. J Appl Dev Psychol.

[45] Blume F, Hudak J, Dresler T, Ehlis A, Kühnhausen J, Renner TJ, Gawrilow C (2017). NIRS-based neurofeedback training in a virtual reality classroom for children with attention-deficit/hyperactivity disorder: study protocol for a randomized controlled trial. Trials.

[46] Chicchi Giglioli IA, de Juan Ripoll C, Parra E, Alcañiz Raya M (2021). Are 3D virtual environments better than 2D interfaces in serious games performance? An explorative study for the assessment of executive functions. Appl Neuropsychol Adult.

[47] Chicchi Giglioli IA, Bermejo Vidal C, Alcañiz Raya M (2019). A virtual versus an augmented reality cooking task based-tools: a behavioral and physiological study on the assessment of executive functions. Front Psychol.

[48] Climent G, Rodríguez C, García T, Areces D, Mejías M, Aierbe A, Moreno M, Cueto E, Castellá J, Feli González M (2021). New virtual reality tool (Nesplora Aquarium) for assessing attention and working memory in adults: a normative study. Appl Neuropsychol Adult.

[49] Coleman B, Marion S, Rizzo A, Turnbull J, Nolty A (2019). Virtual reality assessment of classroom - related attention: an ecologically relevant approach to evaluating the effectiveness of working memory training. Front Psychol.

[50] Dahdah MN, Bennett M, Prajapati P, Parsons TD, Sullivan E, Driver S (2017). Application of virtual environments in a multi-disciplinary day neurorehabilitation program to improve executive functioning using the Stroop task. NRE.

[51] Davison SMC, Deeprose C, Terbeck S (2018). A comparison of immersive virtual reality with traditional neuropsychological measures in the assessment of executive functions. Acta Neuropsychiatr.

[52] De Lillo C, Kirby M, James F (2014). Spatial working memory in immersive virtual reality foraging: path organization, traveling distance and search efficiency in humans (Homo sapiens). Am J Primatol.

[53] Díaz-Orueta U, Garcia-López C, Crespo-Eguílaz N, Sánchez-Carpintero R, Climent G, Narbona J (2014). AULA virtual reality test as an attention measure: convergent validity with Conners' Continuous Performance Test. Child Neuropsychol.

[54] Eom H, Kim KK, Lee S, Hong Y, Heo J, Kim J, Kim E (2019). Development of virtual reality continuous performance test utilizing social cues for children and adolescents with attention-deficit/hyperactivity disorder. Cyberpsychol Behav Soc Netw.

[55] Grewe P, Kohsik A, Flentge D, Dyck E, Botsch M, Winter Y, Markowitsch HJ, Bien CG, Piefke M (2013). Learning real-life cognitive abilities in a novel 360°-virtual reality supermarket: a neuropsychological study of healthy participants and patients with epilepsy. J Neuroeng Rehabil.

[56] Grewe P, Lahr D, Kohsik A, Dyck E, Markowitsch H, Bien C, Botsch M, Piefke M (2014). Real-life memory and spatial navigation in patients with focal epilepsy: ecological validity of a virtual reality supermarket task. Epilepsy Behav.

[57] Henry M, Joyal C, Nolin P (2012). Development and initial assessment of a new paradigm for assessing cognitive and motor inhibition: the bimodal virtual-reality Stroop. J Neurosci Methods.

[58] Huang K (2020). Exergaming executive functions: an immersive virtual reality-based cognitive training for adults aged 50 and older. Cyberpsychol Behav Soc Netw.

[59] Ijaz K, Ahmadpour N, Naismith SL, Calvo RA (2019). An immersive virtual reality platform for assessing spatial navigation memory in predementia screening: feasibility and usability study. JMIR Ment Health.

[60] Iriarte Y, Diaz-Orueta U, Cueto E, Irazustabarrena P, Banterla F, Climent G (2016). AULA-advanced virtual reality tool for the assessment of attention: normative study in Spain. J Atten Disord.

[61] Kang YJ, Ku J, Han K, Kim SI, Yu TW, Lee JH, Park CI (2008). Development and clinical trial of virtual reality-based cognitive assessment in people with stroke: preliminary study. Cyberpsychol Behav.

[62] Liao Y, Chen I, Lin Y, Chen Y, Hsu W (2019). Effects of virtual reality-based physical and cognitive training on executive function and dual-task gait performance in older adults with mild cognitive impairment: a randomized control trial. Front Aging Neurosci.

[63] Lo Priore C, Castelnuovo G, Liccione D, Liccione D (2003). Experience with V-STORE: considerations on presence in virtual environments for effective neuropsychological rehabilitation of executive functions. Cyberpsychol Behav.

[64] Mondellini M, Arlati S, Pizzagalli S, Greci L, Sacco M, Arlati S (2018). Assessment of the Usability of an Immersive Virtual Supermarket for the Cognitive Rehabilitation of Elderly Patients: A Pilot Study on Young Adults.

[65] Neguț A, Jurma A, David D (2017). Virtual-reality-based attention assessment of ADHD: ClinicaVR: Classroom-CPT versus a traditional continuous performance test. Child Neuropsychol.

[66] Nolin P, Martin C, Bouchard S (2009). Assessment of inhibition deficits with the virtual classroom in children with traumatic brain injury: a pilot-study. Stud Health Technol Inform.

[67] Nolin P, Stipanicic A, Henry M, Lachapelle Y, Lussier-Desrochers D, Rizzo A (2016). ClinicaVR: Classroom-CPT: a virtual reality tool for assessing attention and inhibition in children and adolescents. Comput Hum Behav.

[68] Boller B, Corriveau-Lecavalier N, Cloutier S, Belleville S, Ouellet (2018). The virtual shop: a new immersive virtual reality environment and scenario for the assessment of everyday memory. J Neurosci Methods.

[69] Parsons TD, Courtney CG, Dawson ME (2013). Virtual reality Stroop task for assessment of supervisory attentional processing. J Clin Exp Neuropsychol.

[70] Parsons TD, Carlew AR (2016). Bimodal virtual reality Stroop for assessing distractor inhibition in autism spectrum disorders. J Autism Dev Disord.

[71] Parsons T, McMahan T (2017). An initial validation of the Virtual Environment Grocery Store. J Neurosci Methods.

[72] Parsons TD, Courtney CG (2018). Interactions between threat and executive control in a virtual reality Stroop task. IEEE Trans Affective Comput.

[73] Parsons T, Barnett M (2018). Virtual apartment Stroop task: Comparison with computerized and traditional Stroop tasks. J Neurosci Methods.

[74] Parsons TD, Barnett M (2019). Virtual apartment-based Stroop for assessing distractor inhibition in healthy aging. Appl Neuropsychol Adult.

[75] Plechatá A, Sahula V, Fayette D, Fajnerová I (2019). Age-related differences with immersive and non-immersive virtual reality in memory assessment. Front Psychol.

[76] Plotnik M, Doniger G, Bahat Y, Gottleib A, Gal O, Arad E (2017). Immersive Trail Making: Construct Validity of an Ecological Neuropsychological Test.

[77] Proulx C, Cabral A, Choudhury N, Debergue P (2018). Acceptability study of a novel immersive cognitive care platform for remediation of cognitive deficits. Annu Rev CyberTherapy Telemed.

[78] Rizzo A, Buckwalter J, Bowerly T, Van Der Zaag C, Humphrey L, Neumann U, Chua C, Kyriakakis C, Van Rooyen A, Sisemore D (2000). The virtual classroom: a virtual reality environment for the assessment and rehabilitation of attention deficits. CyberPsychol Behav.

[79] Robitaille N, Jackson P, Hébert LJ, Mercier C, Bouyer L, Fecteau S, Richards CL, McFadyen BJ (2017). A virtual reality avatar interaction (VRai) platform to assess residual executive dysfunction in active military personnel with previous mild traumatic brain injury: proof of concept. Disabil Rehabil Assist Technol.

[80] Serino S, Morganti F, Colombo D, Pedroli E, Cipresso P, Riva G (2018). Disentangling the contribution of spatial reference frames to executive functioning in healthy and pathological aging: an experimental study with virtual reality. Sensors (Basel).

[81] Tarnanas I, Mouzakidis C, Schlee W (2013). Functional Impairment in Virtual-Reality-Daily-Living-Activities as a Defining Feature of Amnestic MCI: Cognitive and Psychomotor Correlates.

[82] Tarnanas I, Schlee W, Tsolaki M, Müri R, Mosimann U, Nef T (2013). Ecological validity of virtual reality daily living activities screening for early dementia: longitudinal study. JMIR Serious Games.

[83] Tarnanas I, Tsolaki M, Nef T, M Müri R, Mosimann UP (2014). Can a novel computerized cognitive screening test provide additional information for early detection of Alzheimer's disease?. Alzheimers Dement.

[84] Voinescu A, Fodor L, Fraser D, Mejías M, David D (2019). Exploring the Usability of Nesplora Aquarium, a Virtual Reality System for Neuropsychological Assessment of Attention and Executive Functioning.

[85] Yasuda K, Muroi D, Ohira M, Iwata H (2017). Validation of an immersive virtual reality system for training near and far space neglect in individuals with stroke: a pilot study. Top Stroke Rehabil.

[86] Yeh S, Tsai C, Fan Y, Liu P, Rizzo A (2012). An Innovative ADHD Assessment System Using Virtual Reality.

[87] Yeh S, Chen Y, Tsai C, Rizzo A (2012). An Innovative Virtual Reality System for Mild Cognitive Impairment: Diagnosis and Evaluation.

[88] United Nations Statistical Office (1982). Provisional Guidelines on Standard International Age Classification.

[89] Kolk A, Saard M, Pertens L, Kallakas T, Sepp K, Kornet K (2019). Structured model of neurorehab: a pilot study of modern multitouch technology and virtual reality platforms for training sociocognitive deficit in children with acquired brain injury. Appl Neuropsychol Child.

[90] Rand D, Weiss P, Katz N (2009). Training multitasking in a virtual supermarket: a novel intervention after stroke. Am J Occup Ther.

[91] Howett D, Castegnaro A, Krzywicka K, Hagman J, Marchment D, Henson R, Rio M, King JA, Burgess N, Chan D (2019). Differentiation of mild cognitive impairment using an entorhinal cortex-based test of virtual reality navigation. Brain.

[92] Liao Y, Tseng H, Lin Y, Wang C, Hsu W (2020). Using virtual reality-based training to improve cognitive function, instrumental activities of daily living and neural efficiency in older adults with mild cognitive impairment. Eur J Phys Rehabil Med.

[93] Yoo S, Weatherall A, Wong G, Scott S, Menezes M, Wood N (2019). Clinician Perspective on VR Games for Managing Periprocedural Anxiety in Children.

[94] Biffi E, Maghini C, Marelli A, Diella E, Panzeri D, Cesareo A (2016). Immersive Virtual Reality Platform for Cerebral Palsy Rehabilitation.

[95] Pas E, Johnson S, Larson K, Brandenburg L, Church R, Bradshaw C (2016). Reducing behavior problems among students with autism spectrum disorder: coaching teachers in a mixed-reality setting. J Autism Dev Disord.

[96] Yalon-Chamovitz S, Weiss P (2008). Virtual reality as a leisure activity for young adults with physical and intellectual disabilities. Res Dev Disabil.

[97] Oliveira CR, Lopes Filho BJP, Esteves CS, Rossi T, Nunes DS, Lima MMBMP, Irigaray TQ, Argimon IIL (2018). Neuropsychological assessment of older adults with virtual reality: association of age, schooling, and general cognitive status. Front Psychol.

[98] Liu L, Yu B, Han M, Yuan S, Wang N (2019). Mild cognitive impairment understanding: an empirical study by data-driven approach. BMC Bioinformatics.

[99] Ávila-Villanueva M, Maestú F, Fernández-Blázquez M (2018). Internal consistency over time of subjective cognitive decline: drawing preclinical Alzheimer’s disease trajectories. JAD.

[100] Lauriola M, Mangiacotti A, D'Onofrio G, Cascavilla L, Paris F, Ciccone F, Greco M, Paroni G, Seripa D, Greco A (2018). Late-life depression versus amnestic mild cognitive impairment: Alzheimer's disease incidence in 4 years of follow-up. Dement Geriatr Cogn Disord.

[101] Pimontel MA, Rindskopf D, Rutherford BR, Brown PJ, Roose SP, Sneed JR (2016). A meta-analysis of executive dysfunction and antidepressant treatment response in late-life depression. Am J Geriatr Psychiatry.

[102] Rane LJ, Fekadu A, Papadopoulos AS, Wooderson SC, Poon L, Markopoulou K, Cleare AJ (2012). Psychological and physiological effects of caring for patients with treatment-resistant depression. Psychol Med.

[103] Huygelier H, Schraepen B, van Ee R, Vanden Abeele V, Gillebert CR (2019). Acceptance of immersive head-mounted virtual reality in older adults. Sci Rep.

[104] Roberts AR, De Schutter B, Franks K, Radina ME (2019). Older adults' experiences with audiovisual virtual reality: perceived usefulness and other factors influencing technology acceptance. Clin Gerontol.

[105] Appel L, Appel E, Bogler O, Wiseman M, Cohen L, Ein N, Abrams HB, Campos JL (2019). Older adults with cognitive and/or physical impairments can benefit from immersive virtual reality experiences: a feasibility study. Front Med (Lausanne).

[106] Wellsby M, Pexman PM (2014). Developing embodied cognition: insights from children's concepts and language processing. Front Psychol.

[107] Kuehn E, Perez-Lopez MB, Diersch N, Döhler J, Wolbers T, Riemer M (2018). Embodiment in the aging mind. Neurosci Biobehav Rev.

[108] Islam R, Lee Y, Jaloli M, Muhammad I, Zhu D, Quarles J (2020). Automatic Detection of Cybersickness from Physiological Signal in a Virtual Roller Coaster Simulation.

[109] Magaki T, Vallance M (2019). Developing an Accessible Evaluation Method of VR Cybersickness.

[110] Kim J, Son J, Leem H, Lee S (2019). Psychophysiological alteration after virtual reality experiences using smartphone-assisted head mount displays: an EEG-based source localization study. Appl Sci.

[111] Houzangbe S, Christmann O, Gorisse G, Richir S (2020). Effects of voluntary heart rate control on user engagement and agency in a virtual reality game. Virtual Reality.

[112] Patrão B, Pedro S, Menezes P (2016). Human emotions and physiological signals: a classroom experiment. Int J Onl Eng.

[113] Hinkle L, Khoshhal K, Metsis V (2019). Physiological Measurement for Emotion Recognition in Virtual Reality.

[114] Pinto M, Melo M, Bessa M (2018). Use of the Physiological Response to Improve the Gaming Experience.

